# Gemcitabine enhances cell invasion via activating HAb18G/CD147-EGFR-pSTAT3 signaling

**DOI:** 10.18632/oncotarget.11405

**Published:** 2016-08-19

**Authors:** Bao-Qing Xu, Zhi-Guang Fu, Yao Meng, Xiao-Qing Wu, Bo Wu, Liang Xu, Jian-Li Jiang, Ling Li, Zhi-Nan Chen

**Affiliations:** ^1^ Department of Cell Biology and Cell Engineering Research Center, State Key Laboratory of Cancer Biology, National Key Discipline of Cell Biology, Fourth Military Medical University, Xi'an, China; ^2^ Departments of Molecular Biosciences and Radiation Oncology, University of Kansas, Lawrence, Kansas, USA

**Keywords:** gemcitabine, stress, invasion, HAb18G/CD147, EGFR

## Abstract

Pancreatic cancer, one of the most lethal cancers, has very poor 5-year survival partly due to gemcitabine resistance. Recently, it was reported that chemotherapeutic agents may act as stressors to induce adaptive responses and to promote chemoresistance in cancer cells. During long-term drug treatment, the minority of cancer cells survive and acquire an epithelial-mesenchymal transition phenotype with increased chemo-resistance and metastasis. However, the short-term response of most cancer cells remains unclear. This study aimed to investigate the short-term response of pancreatic cancer cells to gemcitabine stress and to explore the corresponding mechanism. Our results showed that gemcitabine treatment for 24 hours enhanced pancreatic cancer cell invasion. In gemcitabine-treated cells, HAb18G/CD147 was up-regulated; and HAb18G/CD147 down-regulation or inhibition attenuated gemcitabine-enhanced invasion. Mechanistically, HAb18G/CD147 promoted gemcitabine-enhanced invasion by activating the EGFR (epidermal growth factor receptor)-STAT3 (signal transducer and activator of transcription 3) signaling pathway. Inhibition of EGFR-STAT3 signaling counteracted gemcitabine-enhanced invasion, and which relied on HAb18G/CD147 levels. In pancreatic cancer tissues, EGFR was highly expressed and positively correlated with HAb18G/CD147. These data indicate that pancreatic cancer cells enhance cell invasion via activating HAb18G/CD147-EGFR-pSTAT3 signaling. Our findings suggest that inhibiting HAb18G/CD147 is a potential strategy for overcoming drug stress-associated resistance in pancreatic cancer.

## INTRODUCTION

Pancreatic ductal adenocarcinoma (PDAC) is a highly aggressive solid tumor and the fourth cause of cancer-related death [1]. Despite new therapeutic strategies, PDAC has a 5-year survival of less than 5% and a median survival of just over 6 months [2]. Surgical resection is rarely possible for advanced PDAC; thus, the genotoxic DNA-damaging agent gemcitabine has historically been the first-line therapeutic agent. However, most patients with unresectable PDAC either do not respond or respond transiently and modestly to gemcitabine and ultimately die because of therapeutic resistance and subsequent metastatic disease [3]. Therefore, investigating the cellular and molecular mechanisms involved in gemcitabine resistance is urgently needed for developing successful treatments for pancreatic cancer.

Currently, chemo-radiotherapy is the standard cytocidal therapy, which primarily concerns on the ability of a drug to induce cancer cell death. Researchers have focused on developing therapeutic agents that are more effective to eliminate the maximum number of tumor cells [4]. Unfortunately, although great achievements were reached in the beginning, recent studies have revealed that current therapeutic approaches that aim to eradicate the primary tumor may only have short-term benefits and eventually lead to increased tumor cells resistance and metastasis [4, 5]. For example, multiple metastases are induced by the VEGFR kinase inhibitor sunitinib/SU11248 or VEGFR-specific antibodies [6, 7], and the BRAF inhibitor PLX4720 induces metastasis in RAS- or BRAF-mutant melanoma [8]. We have also observed post-radiation tumor recurrence in pancreatic cancer [9]. The failure of current therapeutic approaches is partly due to a lack of understanding of the molecular signaling pathways utilized by cancer cells to actively respond to therapeutic pressures [4, 10]. Accumulating evidence has indicated that chemo-radiotherapy may not kill all the cancer cells but may act as a stressor on the surviving cells, inducing active counter-defense responses that protect cells from drug stress and lead to drug resistance through selection [11, 12]. Thus, elucidating the mechanisms by which tumor cells counteract drug stress may contribute to the development of therapeutic agents that reverse gemcitabine resistance.

Based on the severity and duration of stress, drugs can force cells to adapt, escape, or die. Specifically, cells can succumb to drug stress *via* apoptosis when the stress is harsh and the protective response is unsuccessful (apoptosis), cells can survive and adjust to the original site when the stress is persistent and less severe *via* a series of protective responses (stay and adapt), or cells can move from a hostile niche to a more favorable one when the stress is less severe without eliciting a protective response (avoid and escape) [5, 10]. Due to genetic and epigenetic instability, malignant tumor cells are predisposed to resist drug stress *via* adaptation procedures or stress avoidance mechanisms. Epithelial-mesenchymal transition (EMT), a hallmark of cancer metastasis, is a typical adaptive response to therapeutic induced-DNA damage. EMT influences the cellular sensitivity to gemcitabine and endows pancreatic cancer cells with a drug resistance phenotype [13]. Chemotherapy-induced cell death generally occurs with 48 hours of treatment [14]; however, EMT confers to improved cell survival over a long-term adaptation, which is usually detectable after 3-4 days of treatment. Simply interfering with EMT cannot effectively alter the treatment response, as EMT occurs after tumor cell death decisions are made. Thus, identifying the short-term cellular response to drug stress and determining whether this short-term response promotes chemoresistance in pancreatic cancer are important.

HAb18G/CD147, which belongs to the CD147 (also called EMMPRIN or basigin) family, is a cancer-associated biomarker for detection [15] and an effective target for treatment [16, 17]. Licartin, an antibody against HAb18G/CD147, has been approved to treat primary hepatocellular carcinoma and to prevent tumor recurrence after liver transplantation or radiofrequency ablation in China [16, 17]. Our previous studies have shown that HAb18G/CD147 facilitates cancer metastasis and progression by inducing MMP secretion and cell motility [18] and that HAb18G/CD147 promotes chemoresistance by functioning as a novel unfolded protein response transducer in response to anti-cancer drug-induced cellular stress [19]. HAb18G/CD147 expression also correlates with other cellular stress responses, such as EMT [20], autophagy [21], and anoikis resistance [22, 23], suggesting that HAb18G/CD147 may involve in cellular responses to drug stress. Recently, others and we reported that CD147 is overexpressed in highly aggressive pancreatic cancer and acts as a novel upstream activator in STAT3-mediated pancreatic tumor development [24, 25]. CD147 knock-down *via* RNA interference increases the chemosensitivity of human pancreatic cancer cells to gemcitabine [26]. Anti-CD147 antibodies have been used as positron emission tomography probes for imaging [27] or in gemcitabine-based combination therapy [28] for pancreatic cancer. However, whether HAb18G/CD147 is involved in the short-term stress response of pancreatic cancer cells to gemcitabine is unclear.

This systematic study aimed to investigate the short-term response of pancreatic cancer cells to gemcitabine, to explore the role of HAb18G/CD147 in this response and to determine the corresponding molecular mechanism of action. In response to short-term gemcitabine stress, pancreatic cancer cells accelerate invasion by up-regulating HAb18G/CD147 expression and activating HAb18G/CD147 downstream of EGFR-pSTAT3 signaling. Thus, the activation of early cellular responses protects pancreatic cancer cells from drug stress-induced cell death and may facilitate tumor resistance to therapy. Blocking the short-term response by inhibiting the HAb18G/CD147 signaling pathway may provide a novel therapeutic strategy for overcoming gemcitabine resistance in pancreatic cancer.

## RESULTS

### Gemcitabine enhances the migration and invasion of pancreatic cancer cells

**Figure 1:**
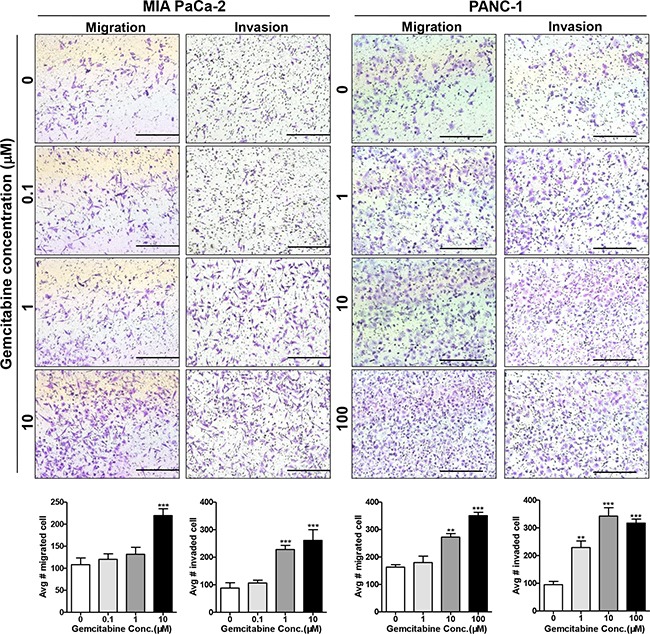
Gemcitabine enhances the migration and invasion of pancreatic cancer cells MIA PaCa-2 and PANC-1 cells were treated with 0, 0.1, 1, 10, or 100 μM gemcitabine for 24 hours and then were seeded on non-coated (migration) and Matrigel-coated (invasion) upper chambers. Cells that migrated/invaded to the lower surface of the filter were fixed, stained, imaged, and counted in 10 random fields. Bars = 500 μm as indicated. **P < 0.01; ***P < 0.001 compared with control.

We first determined the chemo-sensitivity of pancreatic cancer cell lines to gemcitabine, which was assayed by cell growth inhibition at 72 hours. The IC_50_ (drug concentration that caused 50% growth inhibition) values for MIA PaCa-2 and PANC-1 cells were 0.03 ± 0.01 μM and 1.62 ± 0.91 μM, respectively (Supplementary Figure S1A). For an optimal time course of gemcitabine cytotoxicity, we treated MIA PaCa-2 cells for 8-72 hours at concentrations of 0.1–10 μM. The cell relative viability of all dosages was above 80% within 8-24 hours but was below 60% beyond 24 hours (Supplementary Figure S1B). Thus, to explore the short-term cellular response to drug stress, we investigated the migration and invasion by treating cells with 0.1-10 μM gemcitabine for 24 hours. As PANC-1 cells were more resistant to gemcitabine, we increased the minimum and maximum gemcitabine doses to 1 μM and 100 μM. At the 24-hour time point when the cells were analyzed for migration and invasion, the cell viability at different gemcitabine concentrations was above 90% (Supplementary Figure S1C). However, the migration potential of gemcitabine-treated cells increased significantly in a dose-dependent manner compared to untreated cells, with significant increases occurring at 10 μM for MIA PaCa-2 and at 10 μM and 100 μM for PANC-1 cells (Figure 1). A similar increasing trend was also observed in the invasion assay, with more significant enhancement occurring at the same concentration. At 1 μM, gemcitabine did not significantly stimulate migration but did enhance invasion in both PANC-1 and MIA PaCa-2 cells, indicating that invasion potential was a more obvious response to gemcitabine stress than migration. We then adopted *in vitro* invasion assay as a representative means to explore the short-term cellular response to gemcitabine. Together, our results showed that pancreatic cancer cells responded to short-term gemcitabine treatment by enhancing migration and invasion, with more migration and invasion occurring at higher concentration, indicating a potential early active escape mechanism in response to gemcitabine stress.

### HAb18G/CD147 is required for gemcitabine-enhanced migration and invasion in pancreatic cancer cells

**Figure 2:**
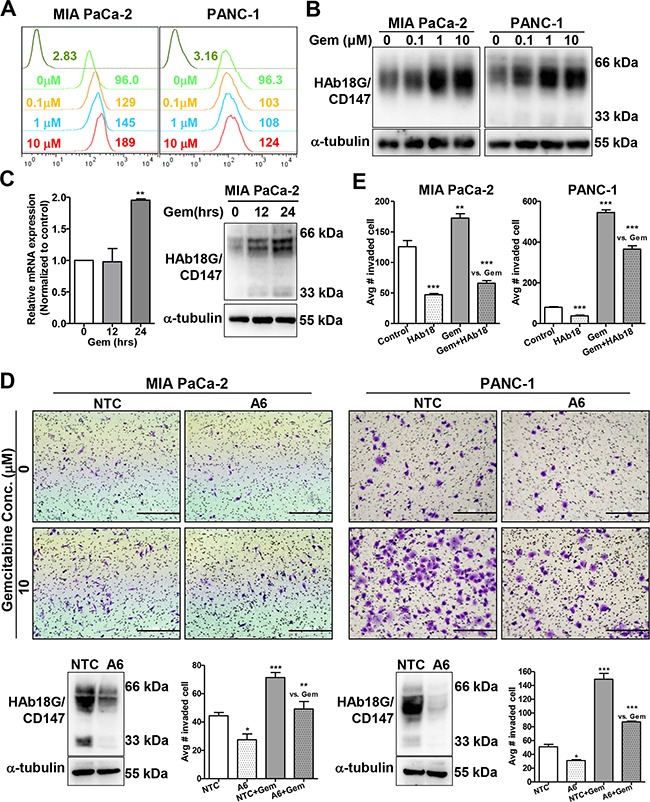
HAb18G/CD147 is required for gemcitabine-enhanced migration and invasion of pancreatic cancer cells **A.** Flow cytometric analysis of the membrane expression of HAb18G/CD147 in pancreatic cancer cells treated with different doses of gemcitabine (0, 0.1, 1, or 10 μM) for 24 hours. Cells were labeled with FITC-conjugated anti-human CD147 antibody, and isotype-matched mouse immunoglobulin was used as a control. **B.** Western blot analysis of HAb18G/CD147 protein levels in pancreatic cancer cells treated with different doses of gemcitabine (Gem) (0, 0.1, 1, or 10 μM) for 24 hours. a-tubulin was included as a loading control. **C.** mRNA (qPCR) and protein (western) levels of HAb18G/CD147 in 10 μM gemcitabine-treated MIA PaCa-2 cells at different time points (0,12, 24 hours). **D.** *In vitro* invasion assay of HAb18G/CD147 knock-down cells treated with or without gemcitabine (10 μM, 24 hours). The photomicrographs at the top illustrate representative fields of invaded cells. Knock-down efficiency was confirmed by immunoblot analysis (bottom left). The number of invaded cells was calculated; the data are presented in a histogram (bottom right). NTC, non-target shRNA control; A6, CD147 shRNA. **E.** *In vitro* invasion assay of pancreatic cancer cells treated with gemcitabine (10 μM, 24 hours) alone or in combination with 30 μg/mL HAb18IgG.

Recently, HAb18G/CD147 was shown to be highly expressed in pancreatic cancer cells; and HAb18G/CD147 is widely involved in metastasis and chemoresistance and correlates with cellular stress responses [24, 26]. Therefore, we explored the role of HAb18G/CD147 in gemcitabine-enhanced migration/invasion by exposing pancreatic cancer cells to 0-10 μM gemcitabine for 24 hours. As shown in Figure 2A and 2B and in Supplementary Figure S2A, gemcitabine up-regulated HAb18G/CD147 protein expression in a dose-dependent manner in both pancreatic cancer cell lines, with the maximal enhancement occurring at 10 μM gemcitabine. The up-regulation of HAb18G/CD147 protein expression was also time-dependent; increased HAb18G/CD147 protein expression occurred as early as 12 hours, before the increases in migration and invasion (Figure 2C). However, HAb18G/CD147 mRNA expression was increased as late as 24 hours, indicating a non-transcription-based mechanism for up-regulating HAb18G/CD147 protein levels at 12 hours.

To further determine whether HAb18G/CD147 is required for gemcitabine-enhanced migration and invasion, we adopted a loss-of-function strategy using the pLKO lentiviral vector [24]. When HAb18G/CD147 expression was effectively silenced (typically 60-80% reduction in protein expression), which was validated by western blot and flow cytometry, gemcitabine-enhanced invasion was significantly attenuated in both PANC-1 and MIA PaCa-2 cells (Figure 2D, Supplementary Figure S2B). Moreover, the anti-HAb18G/CD147 monoclonal antibody HAb18IgG (30 μg/mL) significantly counteracted gemcitabine-enhanced invasion in both cell lines (Figure 2E, Supplementary Figure S2C). These results strongly suggest that HAb18G/CD147 is required for gemcitabine-enhanced migration/invasion in pancreatic cancer cells and that HAb18G/CD147 correlates with the cellular response to gemcitabine stress and the related resistance. HAb18IgG effectively abolished gemcitabine-enhanced migration/invasion, suggesting the therapeutic potential of this antibody in gemcitabine-resistant pancreatic cancer.

### EGFR-STAT3 signaling is involved in gemcitabine-enhanced migration and invasion

**Figure 3:**
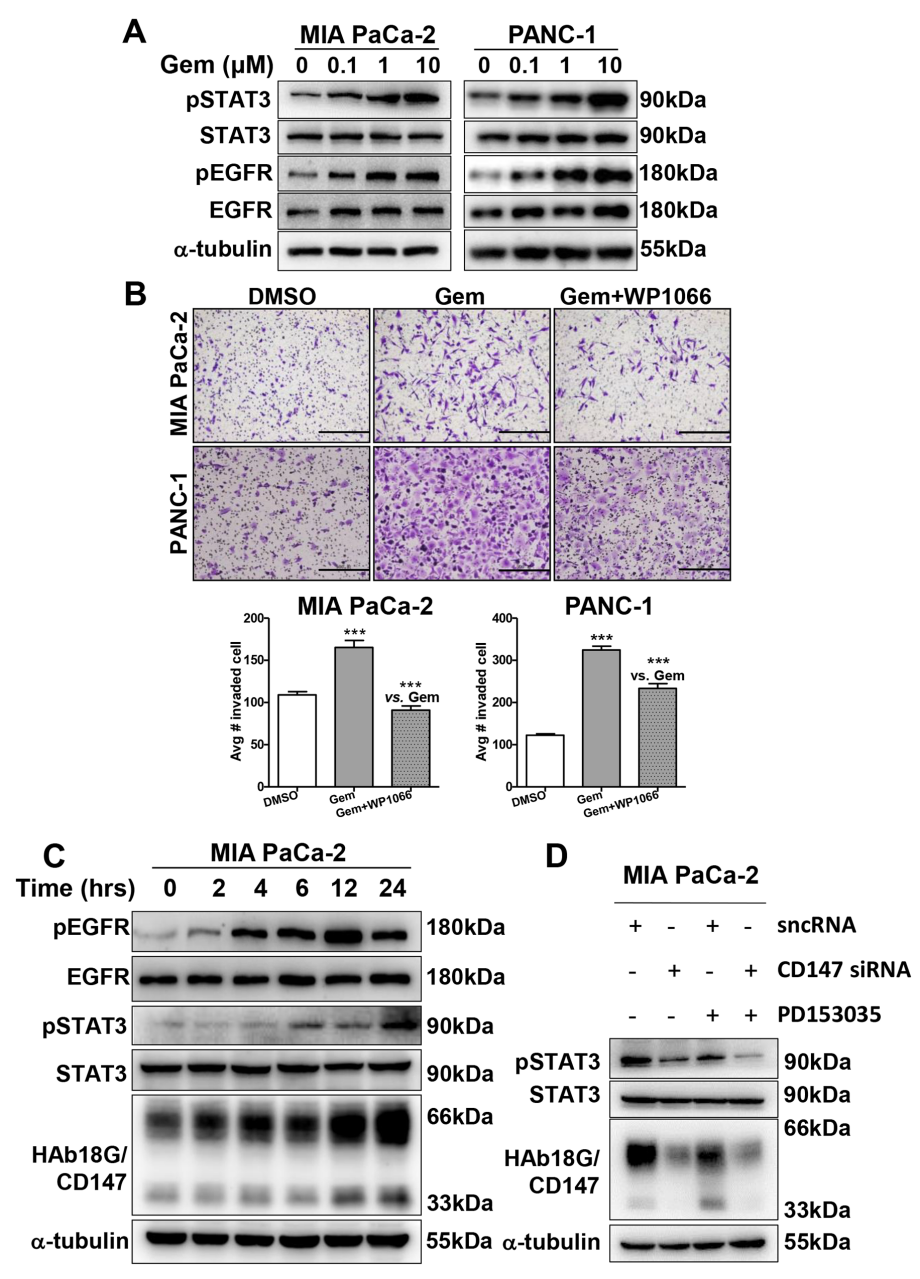
EGFR-STAT3 signaling is involved in gemcitabine-enhanced migration and invasion **A.** Western blot analysis of pSTAT3, STAT3, pEGFR, and EGFR protein levels in pancreatic cancer cells treated with different doses of gemcitabine (0, 0.1, 1, or 10 μM) for 24 hours. **B.** *In vitro* cell invasion of pancreatic cancer cells treated with gemcitabine (10 μM, 24 hours) alone or in combination with WP1066 (1 μM, 24 hours). **C.** Western blot analysis of pSTAT3, STAT3, pEGFR, and EGFR protein levels in 10 μM gemcitabine-treated pancreatic cancer cells at different time points (0, 2, 4, 6, 14, or 24 hours). **D.** Western blot analysis of pSTAT3, STAT3 and CD147 protein levels in pancreatic cancer cells treated with CD147 siRNA and 10 μM PD153035 alone or in combination. Silencer negative control siRNA (sncRNA) was used as a negative control.

We recently reported that HAb18G/CD147 is an upstream activator of STAT3 signaling in pancreatic cancer; the STAT3 pathway is a critical pathway that is activated in gemcitabine-resistant cells [24, 29]. STAT3 activity is also necessary for pancreatic cancer cell invasion via MMP7 [30]. To investigate whether pSTAT3 is also involved in gemcitabine-enhanced migration and invasion in pancreatic cancer cells, we examined phosphorylated STAT3 (pSTAT3) and total STAT3 protein levels after gemcitabine treatment. The protein levels of pSTAT3, but not of total STAT3, increased in a dose-dependent manner in both pancreatic cancer cell lines after 24 hours of gemcitabine treatment (Figure 3A). The pSTAT3-specific inhibitor WP1066 inhibited cell invasion in a dose-dependent manner, with maximal inhibition occurring at 1 μM (Supplementary Figure S3A). Moreover, 1 μM WP1066 significantly abolished gemcitabine-enhanced invasion in both pancreatic cancer cell lines, although the degree of inhibition differed (Figure 3B). By contrast, WP1066 treatment resulted in greater suppression of gemcitabine-related invasion in MIA PaCa-2 cells (45%) than in PANC-1 cells (28%), consistent with the fact that the pSTAT3 expression level is higher in MIA PaCa-2 cells than in PANC-1 cells [24]. Therefore, pSTAT3 appears to be involved in gemcitabine-enhanced migration and invasion of pancreatic cancer cells.

In Figures 1, 2D and 2E, we noticed that gemcitabine-resistant EGFR-mutant PANC-1 cells exhibited greater gemcitabine-enhanced invasion than gemcitabine-sensitive EGFR-wild type MIA PaCa-2 cells, indicating that EGFR might be related to gemcitabine-enhanced invasion. As STAT3 is activated by EGFR [31], we examined whether EGFR is involved in gemcitabine-enhanced invasion by activating STAT3. As shown in Figure 3A, a dose-dependent increase in pEGFR, together with pSTAT3, was observed in 0-10 μM gemcitabine-treated pancreatic cancer cells. However, unlike pEGFR, total EGFR was not changed upon gemcitabine treatment. Moreover, time-dependent increases in pEGFR and pSTAT3 were observed in MIA PaCa-2 cells as early as 4 and 6 hours, respectively; both were later than the increase in CD147, which occurred at 2 hours (Figure 3C). Treating cells with the EGFR inhibitor PD153035 resulted in dose-dependent inhibition of pSTAT3 levels, but not total STAT3 levels (Supplementary Figure S3B), indicating that EGFR is involved in gemcitabine-enhanced invasion *via* STAT3. Thus, the EGFR-STAT3 signaling pathway contributes to gemcitabine-enhanced invasion.

### HAb18G/CD147 activates pSTAT3 signaling via EGFR

As shown above, HAb18G/CD147 and EGFR activate STAT3 signaling, contributing to gemcitabine-enhanced invasion. Therefore, we examined whether HAb18G/CD147 activates gemcitabine-enhanced pSTAT3 signaling *via* EGFR or *vice versa*. As the time-dependent increase in pEGFR occurred later than that in HAb18G/CD147, we deduced that HAb18G/CD147 might activate pSTAT3 signaling *via* EGFR. To elucidate this possibility, we knocked down HAb18G/CD147 expression using siCD147 and then treated cells with or without PD153035. As shown in Figure 3D, pSTAT3 levels greatly decreased in both siCD147- and PD153035-treated cells; and pSTAT3 levels further decreased in response to combined siCD147 and PD153035 treatment, suggesting that the PD153035-induced decrease in the pSTAT3 level correlated with HAb18G/CD147 expression. As the CD147 activation and knock-down influenced the pEGFR levels accordingly (Figure 3D), EGFR-activated pSTAT3 signaling depended on HAb18G/CD147 levels.

**Figure 4:**
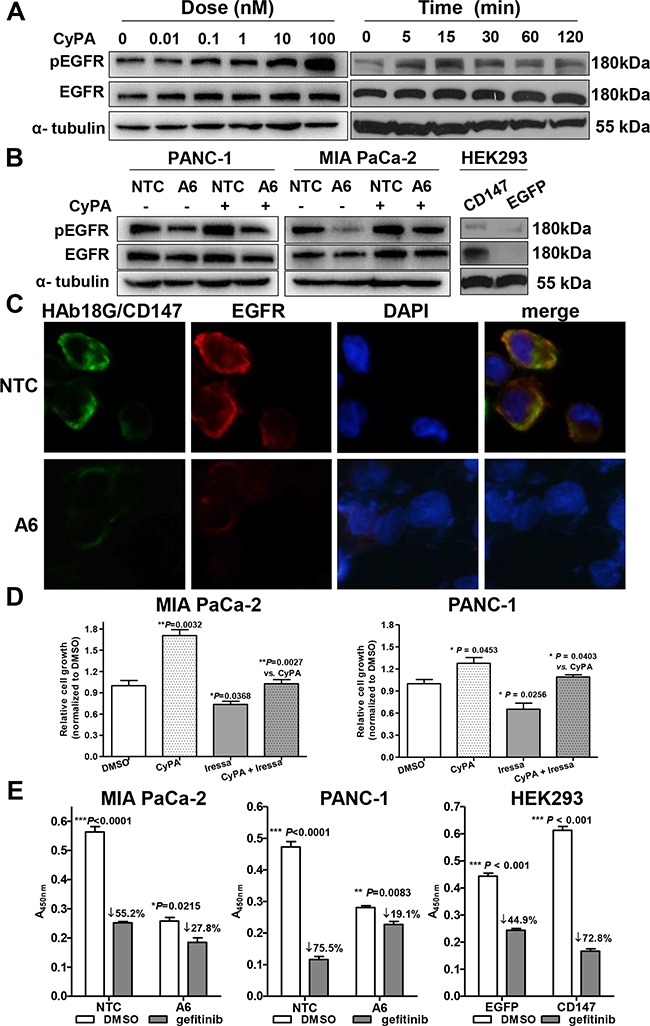
HAb18G/CD147 activates EGFR-pSTAT3 signaling in pancreatic cancer cells **A.** Western blot analysis of pEGFR and EGFR protein levels in serum-starved PANC-1 cells treated with CyPA for different time points (right) at different concentrations (left). Treatment with phosphate-buffered saline was used as a control. **B.** Western blot analysis of pEGFR and EGFR protein levels in serum-starved HAb18G/CD147 knock-down or knock-in cells treated with or without 100 nM CyPA for 30 min. **C.** Immunofluorescence co-labeling of HAb18G/CD147 (green) and EGFR (red) in HAb18G/CD147 knock-down PANC-1 cells. Magnification: 400×. **D.** Cell growth assay in MIA PaCa-2 and PANC-1 cells treated with or without the EGFR inhibitor gefitinib (80 μM, 72 hours) and/or CyPA (100 nM, 30 min). **E.** Cell growth assay in HAb18G/CD147 knock-down or knock-in cells after treatment with the EGFR inhibitor gefitinib (80 μM, 48 hours).

To further explore the relationship between HAb18G/CD147 and EGFR, we tested whether CyPA, a natural ligand for HAb18G/CD147 [24], could up-regulate EGFR levels in pancreatic cancer cells. As shown in Figure 4A, CyPA stimulation caused dose- and time-dependent increases in pEGFR levels. Next, we determined whether the above CyPA effects were HAb18G/CD147 dependent or not by knock-down or knock-in of CD147. Both EGFR and pEGFR protein levels were reduced after HAb18G/CD147 knock-down; moreover, CyPA-induced pEGFR protein expression was attenuated by HAb18G/CD147 knock-down (Figure 4B). In contrast, EGFR and pEGFR protein levels were significantly increased by HAb18G/CD147 knock-in in HEK293 cells, indicating that the CyPA-mediated increase in pEGFR/EGFR protein expression correlated with HAb18G/CD147 levels. Using immunofluorescence staining, we observed that both HAb18G/CD147 and EGFR were evenly distributed on the cell membrane in control vector-transfected PANC-1 NTC cells. However, EGFR membrane expression was significantly reduced after HAb18G/CD147 knock-down in A6 cells (Figure 4C). These results suggest that HAb18G/CD147 activates EGFR protein expression and phosphorylation.

To evaluate whether HAb18G/CD147 functionally influences EGFR in pancreatic cancer cells, we performed cell growth assays after exposing cells to CyPA and the EGFR inhibitor gefitinib. Gefitinib (80 μM) significantly inhibited CyPA-induced cell growth to the level of control in both cell lines (*P* = 0.0027 and *P* = 0.0403 for MIA PaCa-2 and PANC-1, respectively, Figure 4D). Furthermore, the cell growth inhibition by gefitinib correlated with HAb18G/CD147 levels: cell growth decreased 1.96-3.95 fold in cells with HAb18G/CD147 knock-down and increased 1.62-fold in cells with HAb18G/CD147 knock-in (Figure 4E), indicating that EGFR acts downstream of HAb18G/CD147 in pancreatic cancer cells. Together, these results suggest that HAb18G/CD147 contributes to gemcitabine enhanced-invasion *via* activating EGFR-STAT3 signaling.

### HAb18G/CD147 and EGFR are co-overexpressed in human pancreatic cancer

**Figure 5:**
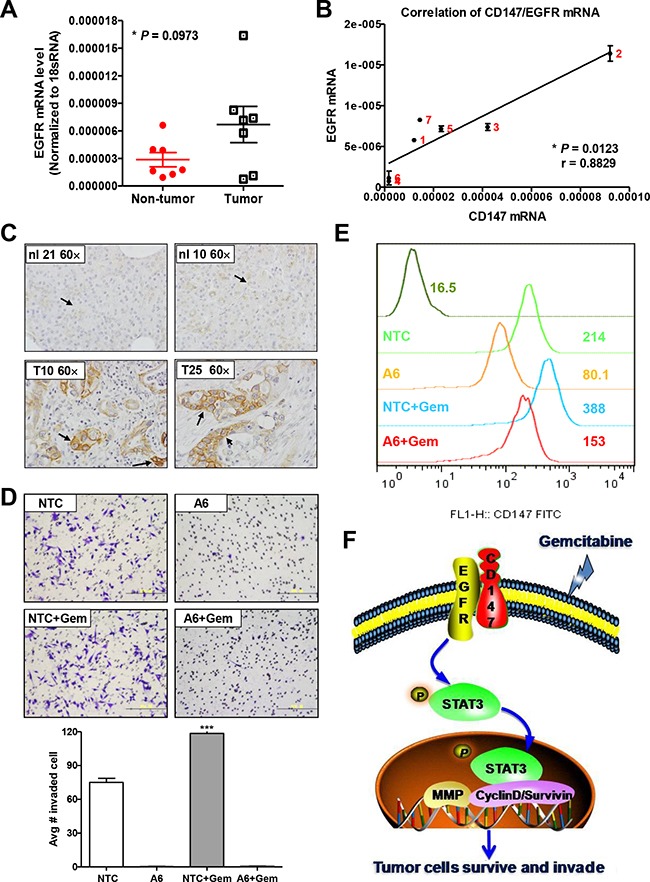
HAb18G/CD147 and EGFR are co-expressed in human pancreatic tumors **A.** EGFR mRNA levels in 7 pairs of PDACs and adjacent non-tumor tissues. Levels were normalized against 18S RNA levels. **B.** Correlation analysis between EGFR and HAb18G/CD147 mRNA expression in 7 PDACs. **C.** Immunohistochemical staining of EGFR protein levels in normal pancreas and PDAC. Representative morphology of EGFR immunostaining in normal pancreatic tissues (No. 10 and No. 21) and in pancreatic cancer tissues (No. 10 and No. 25) is shown. Magnification: 600×. **D.** Boyden chamber assay of cell invasion and **E.** flow cytometric analysis of HAb18G/CD147 membrane expression in tumor cells isolated from mice. **F.** A proposed working model for gemcitabine-enhances pancreatic cancer cell invasion via HAb18G/CD147-EGFR-pSTAT3 signaling.

To investigate whether the expression levels of HAb18G/CD147 and EGFR are associated in human pancreatic cancer, we firstly analyzed EGFR mRNA expression levels in 7 pairs of pancreatic cancer and adjacent non-tumor tissues from patients with pancreatic cancer. As indicated in Figure 5A, EGFR mRNA levels in pancreatic tumor tissues increased 2.33-fold on average compared to those in adjacent non-tumor tissues (*P* = 0.0973, n = 7). Moreover, EGFR mRNA levels significantly correlated with HAb18G/CD147 mRNA levels (Spearman ***r*** = 0.8829, *P* = 0.0123, Figure 5B).

We then analyzed EGFR protein expression in 179 pancreatic tissues. In a pancreatic tissue microarray (TMA) of 179 samples, EGFR was positively expressed in 20.8% (10/48) of normal pancreatic tissues and in 71.4% (5/7), 66.7% (14/21) and 78.6% (81/103) of the chronic pancreatitis, pancreatic preneoplasia and PDAC samples, respectively (Figure 5C, Table 1). The positive ratios of EGFR expression in chronic pancreatitis, pancreatic preneoplasia and PDAC were significantly higher than those in adjacent non-tumor tissues (*P* = 0.019, *P* < 0.001, and *P* < 0.001, respectively). Moreover, the intense staining ratio of EGFR in PDAC (40.8%) increased by 1.43- and 2.15-fold compared with chronic pancreatitis (28.6%) and pancreatic preneoplasia (19.0%), respectively, although these differences did not reach statistical significance.

**Table 1:** EGFR expression in pancreatic tissues

Groups	EGFR expression levels^a^				*P* value^b^
	Low		High		
	0	1	2	3	
**Normal**	79.2% (38/48)	12.5% (6/48)	6.2% (3/48)	**2.1%** (1/48)	**/**
**Pancreatitis**	28.6% (2/7)	42.9% (3/7)	0	**28.6%** (2/7)	**0.019**
**Precancerous lesions**	33.3% (7/21)	28.7% (6/21)	19% (4/21)	**19%** (4/21)	**<0.001**
*PanIN*	*0*	*50%* *(1/2)*	*50%* *(1/2)*	*0*	*N*
*Cystadenoma*	*25%* *(1/4)*	*25%* *(1/4)*	*0*	*50%* *(2/4)*	*N*
*IPMN*	*40%* *(6/15)*	*26.7%* *(4/15)*	*20%* *(3/15)*	*13.3%* *(2/15)*	*0.010*
**PDAC**	21.4% (22/103)	12.6% (13/103)	25.2% (26/103)	**40.8%** (42/103)	**<0.001**

a EGFR expression levels were classified as 0 (no staining), 1 (light staining), 2 (intermediate staining) or 3 (intense staining).

b Estimated using the χ^2^ test compared with normal tissue after dividing the samples into low- and high-expression groups. The low-expression group includes cases with a staining intensity score of 0; the high-expression group includes cases with staining intensity scores of 1-3. N, not detected.

Next, we analyzed the co-expression of EGFR and HAb18G/CD147 in 47 normal pancreas and 102 pancreatic cancer tissues with positive staining for both antigens. As indicated in Table 2, a significantly higher incidence of high expression of both EGFR and HAb18G/CD147 was found in pancreatic cancer tissues compared with normal tissues (71.57% [73/102] *vs*. 17.02% [8/47], *P* < 0.001). The incidence of HAb18G/CD147^high^EGFR^high^ (71.57%) in pancreatic cancer tissues was significantly higher than that of HAb18G/CD147^low^EGFR^low^ (4.90%), HAb18G/CD147^high^ (15.69%), or EGFR^high^ (7.84%) (*P* < 0.001 for all three groups). Furthermore, high HAb18G/CD147 expression was significantly correlated with high EGFR expression (Spearman ***r*** = 0.3086, *P* = 0.0016), suggesting that the high expression levels of both HAb18G/CD147 and EGFR might have important roles in promoting pancreatic cancer progression.

**Table 2:** HAb18G/CD147 and EGFR co-expression in pancreatic tissues

*Group*	*Normal*		*Cancer*	
**CD147^low^EGFR^low^**	5/47	*10.64%*	5/102	*4.90%*
**CD147^high^EGFR^low^**	33/47	*70.21%*	16/102	*15.69%*
**CD147^low^EGFR^high^**	1/47	*2.13%*	8/102	*7.84%*
**CD147^high^EGFR^high^**	8/47	*17.02%*	73/102	71.57%*

Pearson *r* = 0.3086 (95% confidence interval: 0.1157–0.4792), *P* = 0.0016.

* Estimated by χ^2^ test compared with normal pancreas tissues.

### HAb18G/CD147 is essential for gemcitabine-enhanced invasion in mice

Above studies showed that short-term gemcitabine exposure can rapidly enhance *in vitro* cell invasion through up-regulating CD147 expression. Whether this scenario actually existed *in vivo* is unclear yet. Therefore, we examined the response of tumor cells to gemcitabine chemotherapy in mice. MIA PACa-2 NTC or CD147 knock-down A6 cells were inoculated into five- to six-week-old female nude mice subcutaneously. When tumors reached a mean volume of 100mm^3^, we treated tumor-bearing mice with 100mg/kg gemcitabine or saline. 24 hours later, tumor cells were isolated to assess cell invasion. Our results showed that MIA PaCa-2 NTC cells from the gemcitabine-treated mice gained significantly increased invasion ability and significantly increase of CD147 membrane expression when compared with MIA PaCa-2 NTC cells from the vehicle-treated mice (Figure 5D and 5E). On the contrary, CD147 knock-down A6 cells from the gemcitabine-treated mice and the vehicle-treated mice almost lost the invasion ability. This result provides the *in vivo* evidence that gemcitabine-treatment induces an increase of the invasion ability of tumor cells, and HAb18G/CD147 plays an important role in tumor cells evasion of gemcitabine stress *in vivo*.

## DISCUSSION

In this study, we found that pancreatic cancer cells escape short-term gemcitabine-enhanced stress by increasing invasion rather than by succumbing to stress-induced death or *in situ* adaptive survival. In response to short-term gemcitabine stress, HAb18G/CD147 was up-regulated and promoted invasion. Inhibition or blocking HAb18G/CD147 *via* knock-down or antibody treatment attenuated gemcitabine-enhanced invasion. We also found that the EGFR-STAT3 signaling pathway was activated in response to short-term gemcitabine treatment and that inhibition of EGFR-STAT3 signaling counteracted gemcitabine-enhanced invasion, which correlated with HAb18G/CD147 levels. In pancreatic cancer tissues, high HAb18G/CD147 expression was significantly correlated with high EGFR expression, and high expression of both HAb18G/CD147 and EGFR was observed in 71.57% of pancreatic cancer samples. Taken together, these data revealed that pancreatic cancer cells actively defend against gemcitabine stress by accelerating cell invasion *via* activating HAb18G/CD147-EGFR-pSTAT3 signaling. An antibody against HAb18G/CD147 could be a potential therapeutic agent for overcoming gemcitabine stress-associated resistance in pancreatic cancer.

Our results and others' show that current therapies, such as the genotoxic DNA-damaging agent gemcitabine, show efficacy in early stages of cancer but may increase oncogenic characteristics such as invasiveness and metastasis later on [32]. Normally, the response to therapy (inhibition of growth and/or survival of cancer cells) and selection for resistance will occur simultaneously [33]. While therapy-induced killing of cancer cells results in therapeutic response and cancer remission, they simultaneously select for drug resistance [11, 12]. These resistant cells proliferate, forming a resistant tumor in relapse, accompanying by tumor progression [33]. Actually, anticancer drugs may promote the emergence of resistance and tumor recurrence [4, 12]. Thus, in addition to drug cytotoxicity, the cellular responses to a given drug must also be considered. We need to reach a balance between the cellular response to a drug and the cytotoxic effects of a drug. Our results clearly illustrate this point by revealing that gemcitabine enhanced pancreatic cancer cell invasion and migration in a dose-dependent manner, with greater invasion and migration in response to higher doses of gemcitabine. The drug stress stimulated invasion was altered according to the intensity of the drug-related stress. Hence, elucidating the mechanisms to counteract the early responses of tumor cells to drug stress may provide strategy for suppressing drug stress-associated resistance while still attacking the cells and may provide further predictive information for determining the response of tumor cells to a particular drug.

Tumor cells evade or adapt in response to drug stress. In this study, we observed increased cell invasion after gemcitabine treatment for 24 hours, which enabled cancer cells to escape drug-induced cell death; however, we did not observe a typical change of EMT markers expression (Supplementary Figure S4B). Our results revealed another mechanism of stress-associated drug resistance that results from a short-term stress escape response; the increased invasion capability was the cause, not the result, of drug resistance. Similar results were also reported by Arora et al., who found that both the up-regulation of CXCR4 expression and the subsequent increased invasiveness served as counter-defense mechanisms against gemcitabine [32]. These protective evasion responses permit short-term cancer cell survival before the onset of cell death and extend cell viability if the environment becomes permissible. However, whether the migrated cells can resist further long-term drug treatment is unclear. Furthermore, the molecular circuits that connect the cross-talk between early stress responses and long-term adaptation remain poorly understood.

Gemcitabine, a genotoxic DNA-damaging agent, directly incorporates into DNA or inhibits ribonucleotide reductase to prevent DNA replication, thus inducing cell death and cell cycle arrest. Moreover, gemcitabine has been reported to inhibit pancreatic cell invasion at a low dose of 50 nM [34, 35] or have no effect at a dose of 100 nM [36, 37]. However, gemcitabine at a higher dose of 10 μM *in vitro* or in a bolus injection at the maximum tolerated dose of 500 mg/kg *in vivo* facilitated cell invasion and accelerated metastasis in mice, respectively [32, 38, 39]. These results suggest that the cellular response to short-term, high-dose chemotherapy promotes invasion and metastasis, which is consistent with our present results. In our study, PANC-1 and MIA PaCa-2 cells were treated with 1-10 μM gemcitabine, which significantly increased the invasion capacities of both cell lines. The doses of 1-10 μM are significantly higher than the IC_50_ of gemcitabine in MIA PaCa-2 cells but are equal to the IC_50_ in PANC-1 cells. A relatively lower dose of gemcitabine was used in PANC-1 cells than in MIA PaCa-2 cells, but a greater increase in invasion was observed in PANC-1 cells. The different genetic backgrounds of the two cell lines potentially explain this difference, *i.e*., PANC-1 cells express mutated EGFR, whereas EGFR is wild type in MIA PaCa-2 cells. Taken together, both a higher dose of gemcitabine and the genetic background of the cells contribute to the *in vitro* active cellular stress response of increased invasion.

We previously showed that the cell adhesion molecule HAb18G/CD147 stimulates cellular stress responses, such as unfold protein response [19], EMT [20], autophagy [21], and anoikis resistance [22, 23]. In this paper, we report that HAb18G/CD147 is up-regulated in response to drug stress and is required for gemcitabine-enhanced invasion. Furthermore, rather than being a cell death signal, HAb18G/CD147 up-regulation was a protective pro-survival response. In addition to cancer, CD147 has been reported to protect neurons against *in vitro* cholesterol and amyloid-b stress [40], oxidative and ischemic injury [41] and focal cerebral ischemia [42]. All these studies suggest that CD147 has a cytoprotective role in response to various injuries, including drug stress and that CD147 may be a stress response protein. To the best of our knowledge, the present study is the first to demonstrate that CD147 functions in chemoresistance partly by actively responding to cellular stress, in addition to its roles in regulating hyaluronan signaling [43] and ABCG2 cellular localization and dimerization [44].

Regarding the mechanism of CD147 up-regulation, our previous publication demonstrated that the HAb18G/CD147 promoter contains a hypoxia-inducible factor response element, which enables increased transcription in response to hypoxia [45]. However, in our present study, the up-regulation of CD147 occurred at the protein level, specifically at the membrane protein level, indicating a completely different scenario. As the up-regulation of CD147 occurred as early as 2 hours post-gemcitabine treatment, we can reasonably infer that the rapid recycling of CD147 to the plasma membrane and impaired degradation may contribute to this rapid induction [46]. Consequently, CD147 can respond quickly to drug stress by influencing cell adhesion and migratory properties. The detailed mechanism by which CD147 is rapidly up-regulated remains under active investigation.

We previously reported that HAb18G/CD147 promotes pSTAT3-mediated pancreatic cancer progression *via* CD44s [24]. In this study, we show that HAb18G/CD147 contributes to gemcitabine stress-enhanced invasion by activating pSTAT3, indicating a novel role for CD147 in STAT3-mediated chemoresistance in addition to promoting PDAC. Among the STAT3 upstream membrane proteins that may associate with CD147, we determined that EGFR was activated in response to short-term gemcitabine treatment. Therefore, we propose that in response to gemcitabine stress, pancreatic cancer cells first up-regulate HAb18G/CD147 expression and then activate EGFR-STAT3 signaling *via* phosphorylation at tyrosine 705 to promote the transcription of STAT3 target genes, such as MMP and cyclin D1/survivin, and ultimately to increase cell survival and invasion (Figure 5F). Our previous findings and literature both suggest that HAb18G/CD147 activated EGFR signaling in pancreatic cancer cells may depend on CD44 [24, 47]. For the cellular response to gemcitabine stress, furthermore, we actually observed a slightly increase of CD44 expression upon gemcitabine treatment, as shown in Supplementary Figure S4A. Therefore, the activation of EGFR signaling by CD147 in gemcitabine-enhanced invasion process possibly depended on CD44 expression.

In human pancreatic cancer, high EGFR expression is required for both the initiation and survival of ADM (and pancreatic intraepithelial neoplasia [PanIN]) lesions, and EGFR ablation restricts the development of PDAC [48]. Inhibiting EGFR signaling has also been reported to improve the efficacy of gemcitabine in human pancreatic tumor xenograft models [49]. Erlotinib, an EGFR kinase inhibitor, sensitizes metastatic pancreatic cancer patients to gemcitabine; the combination of erlotinib and gemcitabine was approved as a therapy for PDAC based on a survival benefit of approximately two weeks [50]. However, only limited efficacy was shown [51]. Our study may provide a strategy for counteracting gemcitabine stress-associated resistance by inhibiting EGFR upstream signaling.

In conclusion, our study suggests that pancreatic cancer cells actively respond to short-term gemcitabine stress by inducing invasion *via* up-regulating HAb18G/CD147 and activating downstream EGFR-pSTAT3 signaling. Our results will be valuable for obtaining a better understanding of the flexibility and interplay of the balanced biosystem between tumor cells and drug stress and the powerful potential of tumor cells to adapt to environmental stress. In addition, our results establish that inhibiting HAb18G/CD147 before gemcitabine treatment may provide a novel combination strategy to overcome gemcitabine stress-associated resistance in pancreatic cancer.

## MATERIALS AND METHODS

### Antibodies, drugs and reagents

The following antibodies were used in this study: anti-EGFR (Cell Signaling Technology, Danvers, MA), anti-STAT3 and anti-vimentin (Proteintech Group Inc, Chicago, IL), anti-phospho-EGFR (pY1173) and anti-phospho-STAT3 (pY705) (Epitomics, Burlingame, CA), anti-CD44, anti-SNAI1 and anti-α-tubulin (Santa Cruz Biotechnology, Dallas, TX), anti-N-cadherin, goat anti-mouse DyLight 488 and goat anti-rabbit DyLight 488 (Thermo Fisher Scientific, Rockford, IL), PE-conjugated anti-human CD147 and isotype-matched mouse immunoglobulin (Miltenyi Biotec, Auburn, CA), and goat anti-rabbit Texas-Red and goat anti-mouse fluorescein isothiocyanate (FITC) (Jackson ImmunoResearch, West Grove, PA). Goat anti-rabbit horseradish peroxidase (HRP), goat anti-mouse HRP, mouse IgG, geneticin (G418) and Lipofectamine 2000 transfection reagent were purchased from Invitrogen (Carlsbad, CA). The anti-mouse HAb18G/CD147 antibody HAb18IgG was prepared as previously reported [16].

Recombinant human CyPA was purchased from Sigma-Aldrich (St. Louis, MO), puromycin was purchased from InvivoGen (San Diego, CA), WP1066 was obtained from Calbiochem (Billerica, MA), gemcitabine was purchased from Lilly France S.A. (Fegersheim, France), and PD153035 and gefitinib were obtained from Selleck (Boston, MA).

### Cell lines and constructs

The human pancreatic cancer cell lines PANC-1, MIA PaCa-2 and the embryonic kidney cell line HEK293 were obtained from American Type Culture Collection and were cultured in DMEM (HyClone, Logan, UT) supplemented with 10% fetal bovine serum (FBS, HyClone, Logan, UT). CD147 pLKO.1 lentiviral shRNA (A6) was obtained from Open Biosystems. The MISSION® Non-Target shRNA Control Vector (pLKO.1-NTC) was obtained from Sigma-Aldrich. Human CD147 cDNA was subcloned into the pEGFP-N1 expression vector (Clontech, Mountain View, CA) as described previously [24].

### Establishment of stable cell lines

CD147 lentiviral shRNA or non-target control shRNA was introduced into cells using FuGENE 6 (Roche). CD147/EGFP cDNA and pEGFP control vectors were transfected into HEK293 cells using Lipofectamine 2000 (Invitrogen) [24]. Knock-down or knock-in cells were selected by adding 4-6 μg/mL puromycin or 1 mg/mL G418 to the culture medium. The silencing or up-regulation of HAb18G/CD147 expression was verified by qPCR, immunoblot and flow cytometry.

### RNA interference

Chemically synthesized, double-stranded CD147 siRNAs were purchased from GenePharma Co., Ltd (Shanghai, China). The sequence for CD147 siRNA (siCD147) is 5′- GUUCUUCGUGAGUUCCUCtt-3′ [18]. Silencer negative control siRNA (sncRNA) was used as a negative control.

### Cell growth assay

Cells were plated in 24- or 96-well plates; cell viability was determined by using a hemocytometer or by measuring WST-8 dye absorbance at 450 nm. For cytotoxicity assays, cells were exposed to different concentrations of gemcitabine. Chemo-sensitivity values were expressed as IC_50_ values. To determine the growth inhibition effect of the EGFR inhibitor gefitinib, cells were serum-starved for 24 hours before the addition of 80 μM gefitinib for one hour followed by a 72-hour treatment with 100 nM CyPA. The results are presented as relative cell growth inhibition normalized to their individual controls. To determine the effect of CD147 knock-down or knock-in on gefitinib-mediated growth inhibition, NTC or A6 cells were treated with 80 μM gefitinib for 48 hours and then analyzed. The cell growth inhibition ratio of gefitinib is indicated with an arrow in the figures.

### *In vitro* migration and invasion assay

*In vitro* migration and invasion assays were performed using 24-well BioCoat Matrigel Invasion Chambers (BD Biosciences Cat No. 354480) containing BD Falcon Cell Culture Inserts with an 8-μm-diameter pore size PET membrane that were coated without (migration) or with (invasion) Matrigel matrix. To perform the assays, the inserts containing Matrigel were hydrated using 500 μL of warm culture medium without serum at 37°C for 2 hours. After hydration, the medium was removed from the chambers, and the inserts were placed on top of each well containing 3-8×10^4^ cells pre-treated with gemcitabine or WP1066. Media containing 10% FBS was added to the lower chamber, and the cells were incubated for 16 hours. Cells that remained on the upper surface of the insert membrane were completely removed with a cotton swab. Cells that had migrated or invaded through the membrane/Matrigel to the bottom of the insert were fixed, stained with 0.2% crystal violet and imaged. The invasive potential of the cells was determined by counting the number of cells that had invaded to the lower surface of the filter in 10 different areas using a Nikon ECLIPSE Ti inverted light microscope (Pudong New District, Shanghai, China). Each assay was performed in triplicate in three separate experiments.

### Flow cytometry analysis

For the flow cytometric analysis, 10^6^ cells were incubated with FITC-conjugated anti-human CD147 antibody in Hank's Balanced Salt Solution (HBSS, Gibco) containing 2% FBS at 4°C for 30 min. Isotype-matched mouse immunoglobulin served as the control. Samples were analyzed using a FACS Calibur Flow Cytometer and CellQuest software (BD Biosciences, San Jose, CA).

As for flow cytometry analysis of EMT marker staining, cells with or without gemcitabine treatment were blocked with 1% BSA in PBS on ice for 30 min. The cells were then incubated on ice with individual antibody N-cadherin (1:100), SNAI1 (1:100), Vimentin (1:100) for 1 h. After washing twice with PBS, goat anti-mouse DyLight 488 or goat anti-rabbit DyLight 488 at a dilution of 1:200 were incubated for another 30 min. Finally, cells were suspended in PBS containing 2% FBS, and analyzed.

### Western blot analysis

Whole cell extracts from cultured cells were prepared by adding phos-RIPA lysis buffer (1 M Tris-HCl (pH 7.5), 5 M NaCl, 0.01% NP-40, 0.5 M EGTA, and 10% SDS) supplemented with Halt phosphatase inhibitor (Thermo Fisher Scientific, Rockford, IL) and complete protease inhibitor cocktail (Roche, Indianapolis, IN) to the cell monolayer. Proteins were separated on 10% polyacrylamide gels and transferred to PVDF membranes. Membranes were blocked in 5% skim milk for 1 hour. Subsequently, the membranes were incubated with each individual antibody overnight at 4°C and then with horseradish peroxidase-conjugated anti-mouse or anti-rabbit IgG for 2 hours. Finally, signals were developed using SuperSignal West Pico Chemiluminescent Substrate (Thermo Fisher Scientific, Rockford, IL). To demonstrate equal loading, membranes were stripped and reprobed with a monoclonal antibody against b-actin or a-tubulin.

### Quantitative real-time PCR (qPCR)

Total RNA extraction and cDNA synthesis were performed as previously described [9]. qPCR amplification was performed using the Stratagene Mx3005P Multiplex quantitative PCR system (Agilent Technologies, Santa Clara, CA) with gene-specific primers for HAb18G/CD147: *forward*: 5′-TCGCGCTGCTGGGCACC-3′; *reverse*: 5′-TGGCGCTGTCATTCAAGGA-3′. Genes of interest were normalized to the housekeeping gene 18S RNA: *forward*: 5′-CGCCGCTAGAGGTGAAATTC-3′; *reverse*: 5′-TTGGCAAATGCTTTCGCTC-3′. Relative mRNA levels are presented as unit values of 2^−ΔCt^ = 2^−(Ct (HKG) − Ct (GOI))^.

### Immunofluorescence staining

Cells grown on chambered cover slips were fixed, blocked and probed with the anti-HAb18G/CD147 antibody HAb18IgG and anti-EGFR antibody. The signals were detected with fluorochrome-conjugated FITC and Texas Red. Cover slips were counterstained with 4′,6-diamidino-2-phenylindole (DAPI, Invitrogen) to visualize the nuclei. Cell images were observed and acquired using a fluorescence microscope (Nikon ECLIPSE Ti, Pudong New District, Shanghai, China).

### Immunohistochemistry staining

TMA staining was performed using standard immunohistochemical staining procedures. To confirm the specificity of the primary antibodies, tissue sections were incubated with control mouse IgG in the absence of primary antibody. The number of positively stained cells and the intensity of positive staining on epithelium cells were independently scored by 2 pathologists in a blinded manner. The percentage of positive cells was scored as follows: 0, 1-25%, 26-75% and > 75%. The intensity of positive immunostaining was graded by an experienced pancreatic pathologist in a blinded manner and was classified into four categories: 0 (no visible staining), 1 (light brown staining), 2 (medium brown staining), and 3 (dark brown staining), with the same intensity covering more than 75% of the stained area. For tissue samples with surface-bound HAb18G/CD147 and EGFR, samples with no staining were classified as negative or no staining; samples with 1+ staining in ≤ 50% of cells or 2+ staining in ≤ 25% of cells were classified as light staining; samples with 1+ staining in > 50% of cells, 2+ staining in 26-75% of cells or 3+ staining in ≤ 25% of cells were classified as intermediate staining; and samples with 2+ staining in > 75% of cells or 3+ staining in > 25% of cells were classified as intense staining. For the statistical analysis, the stained tumor tissues were divided into two groups: the low-expression group and the high-expression group. The low-expression group included cases with a negative staining intensity score; the high-expression group included cases with staining intensity scores of light staining, intermediate staining and intense staining.

### Patient samples

Fresh and paraffin-embedded pancreatic tumor and adjacent non-tumor tissues from pancreatic cancer patients were obtained from the University of Michigan Comprehensive Cancer Center (UMCCC) Histology Core according to an IRB-approved human protocol (H7094). TMAs from 179 patient tissues were obtained from the UMCCC Histology Core for the analysis of HAb18G/CD147 and EGFR expression. Two different recipient paraffin blocks were generated: “control tissue array” and “tumor tissue array”. The “control tissue array” included 48 cases of normal pancreatic tissue and 7 cases of pancreatitis; the “tumor tissue array” included 2 cases of PanIN, 4 cases of cystadenoma, 15 cases of intraductal papillary mucinous neoplasm (IPMN) and 103 cases of PDAC.

### Animal studies

All animal experiments were performed in accordance with Institutional Animal Care and Use Committee approved protocols. For the localized subcutaneous xenograft mouse model, 1×10^6^ MIA PACa-2 NTC or CD147 knock-down A6 cells in 0.2mL DMEM were inoculated into five- to six-week-old female nude mice subcutaneously. When tumors reached a mean volume of 100mm^3^, mice were randomized into four groups (n=5 mice per group). Tumor volume was measured using vernier calipers twice a week and the tumor volume was calculated using the formula described by us previously [24]. Gemcitabine (100 mg/kg) or saline (control group) was administered as a single intraperitoneal injection. 24 hours later, the subcutaneous tumors were harvested from the flanks of the mouse by blunt dissection. Tumors were mechanically minced and incubated at 37°C for 2 hours in the cell dissociation buffer (DMEM supplemented with 10% FBS, penicillin-streptomycin (Invitrogen, Carlsbad, CA), 200 U/mL collagenase type IV (Invitrogen, Carlsbad, CA), and 0.6 U/mL dispase (Sigma-Aldrich, St. Louis, MO). The cell suspension was then passed through a 70-μm filter (BD Biosciences, San Jose, CA) and the cells were collected for the invasion assay [52].

### Statistical analysis

All the data are presented as the mean ± SD of triplicate values from three separate experiments. Independent Student's *t* tests or one-way ANOVAs were used to compare the continuous variables between 2 groups or more than 2 groups, and categorical variables were compared using the χ^2^ test. Spearman rank correlation was conducted to analyze the correlation between the expression of HAb18G/CD147 and EGFR mRNA. Statistical analyses were performed using SPSS 13.0 (IBM) and Prism 5.0 (GraphPad). *P < 0.05 was considered statistically significant.

## SUPPLEMENTARY FIGURES


