# Supplementary material for: Gemcitabine enhances cell invasion via activating HAb18G/CD147-EGFR-pSTAT3 signaling
Source: Oncotarget. 2016 Aug 19;7(38):62177–93. doi: 10.18632/oncotarget.11405 (PMC5308719; doi:10.18632/oncotarget.11405)
Supplement: Supplementary file 1 [file oncotarget-07-62177-s001.pdf]

# Gemcitabine enhances cell invasion via activating HAb18G/CD147-EGFR-pSTAT3 signaling

## SUPPLEMENTARY FIGURES

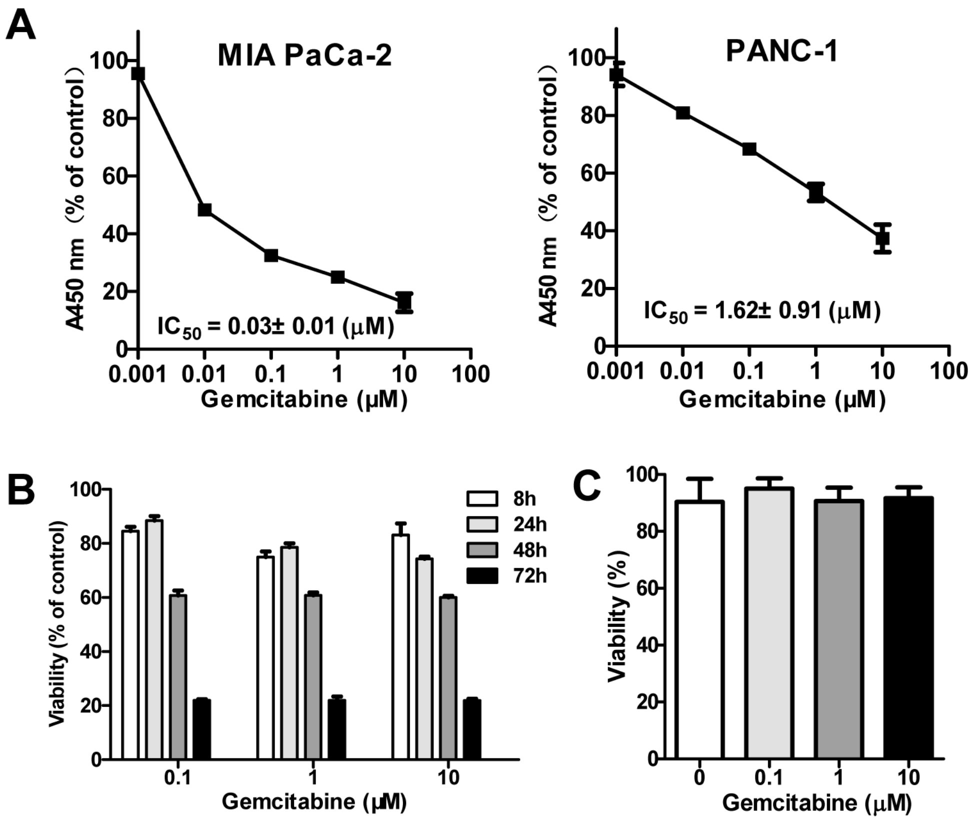


**Supplementary Figure S1: Gemcitabine enhances the migration and invasion of pancreatic cancer cells. A.** Chemosensitivity of MIA PaCa-2 cells and PANC-1 cells to gemcitabine. $IC_{50}$ values were calculated at 72 hours by fitting sigmoidal dose-response curve using GraphPad Prism version 5.0. **B.** Relative cell viability after treatment with various concentrations of gemcitabine (0, 0.1, 1, or 10 μM) for different time points (8 to 72 hours) was examined by WST-8 assay. MIA PaCa-2 cells treated with PBS were used as control. **C.** Cell viability measurement by hemocytometer. MIA PaCa-2 cells were treated with gemcitabine for 24 hours at various concentrations (0, 0.1, 1, or 10 μM) and the cell viability was examined immediately.

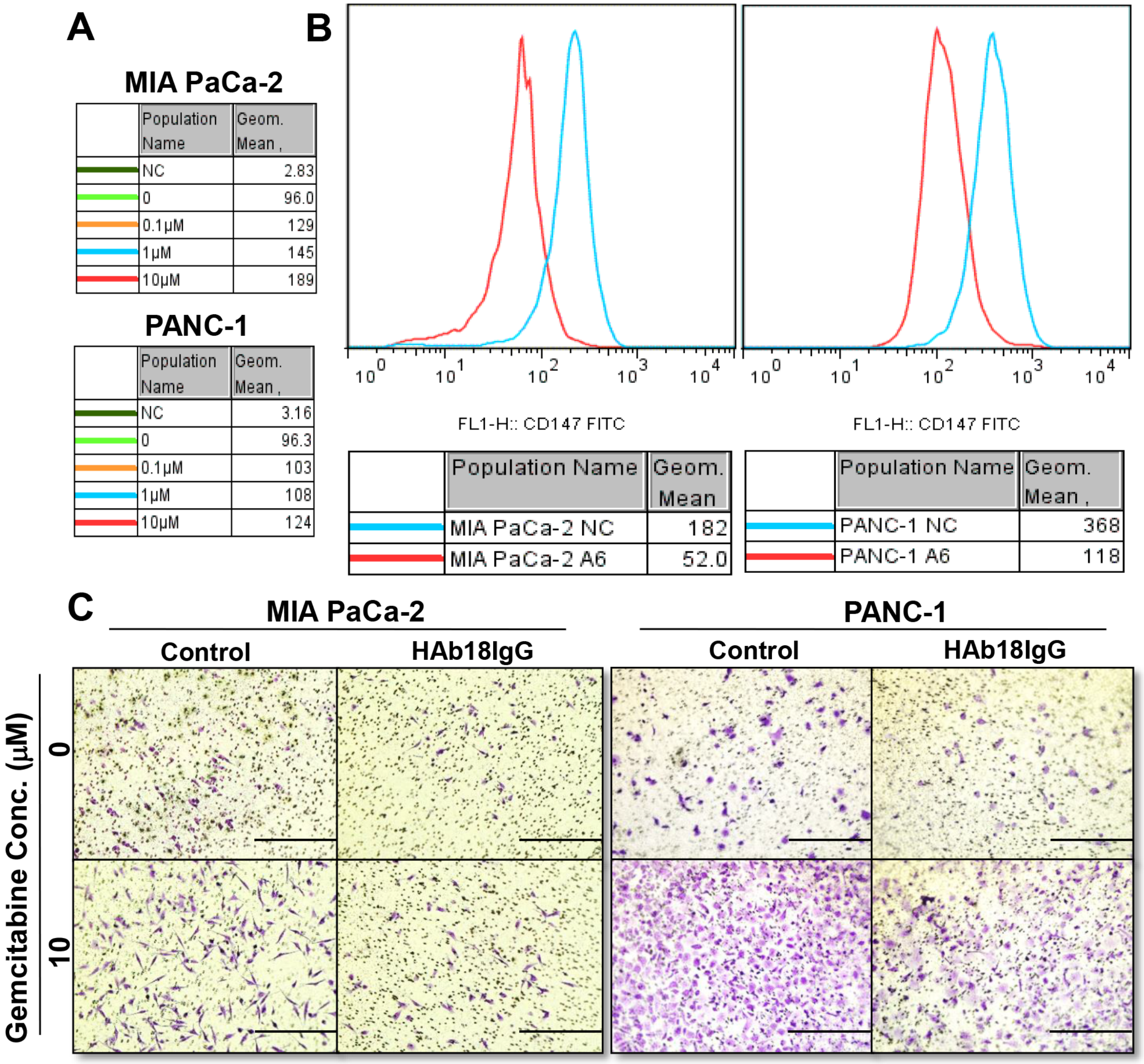


**Supplementary Figure S2: HAb18G/CD147 is required for gemcitabine-enhanced invasion of pancreatic cancer cells.** Cells were labeled with FITC-conjugated anti-human CD147 antibody, and isotype-matched mouse immunoglobulin was used as a control. **A.** Flow cytometry analysis of the HAb18G/CD147 membrane levels in pancreatic cancer cells treated with different doses of gemcitabine (0, 0.1, 1, or 10 µM) for 24 hours. **B.** Flow cytometry analysis of the membrane levels of HAb18G/CD147 in CD147 knock-down cells. NC, non-target shRNA control; A6, CD147 shRNA. **C.** *In vitro* invasion assay of pancreatic cancer cells treated with gemcitabine (10 µM, 24 hours) alone or in combination with 30 µg/ml HAb18IgG. Cells that migrated/invaded to the lower surface of the filter were fixed, stained, imaged, and counted in 10 random view fields. Bars = 500 µm as indicated. **$P$< 0.01; ***$P$< 0.001 compared with control.

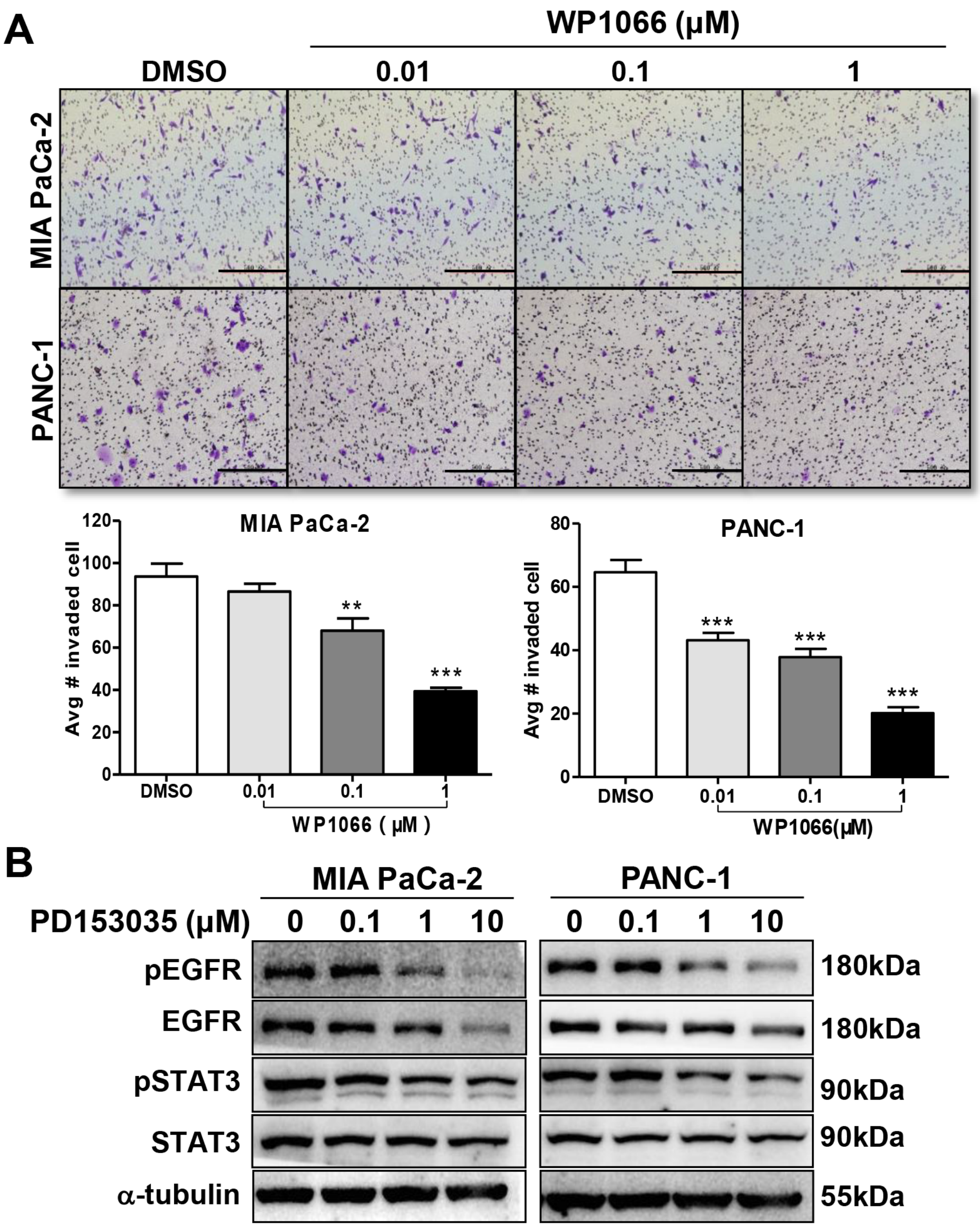


**Supplementary Figure S3: EGFR-STAT3 signaling is involved in gemcitabine-enhanced invasion. A.** *In vitro* invasion of pancreatic cancer cells treated with different doses of WP1066 (0, 0.01, 0.1, or 1 µM) for 24 hours. **B.** Western blot of pSTAT3, STAT3, pEGFR, and EGFR expression in pancreatic cancer cells treated with different doses of PD153035 (0, 0.1, 1, or 10 µM) for 24 hours.

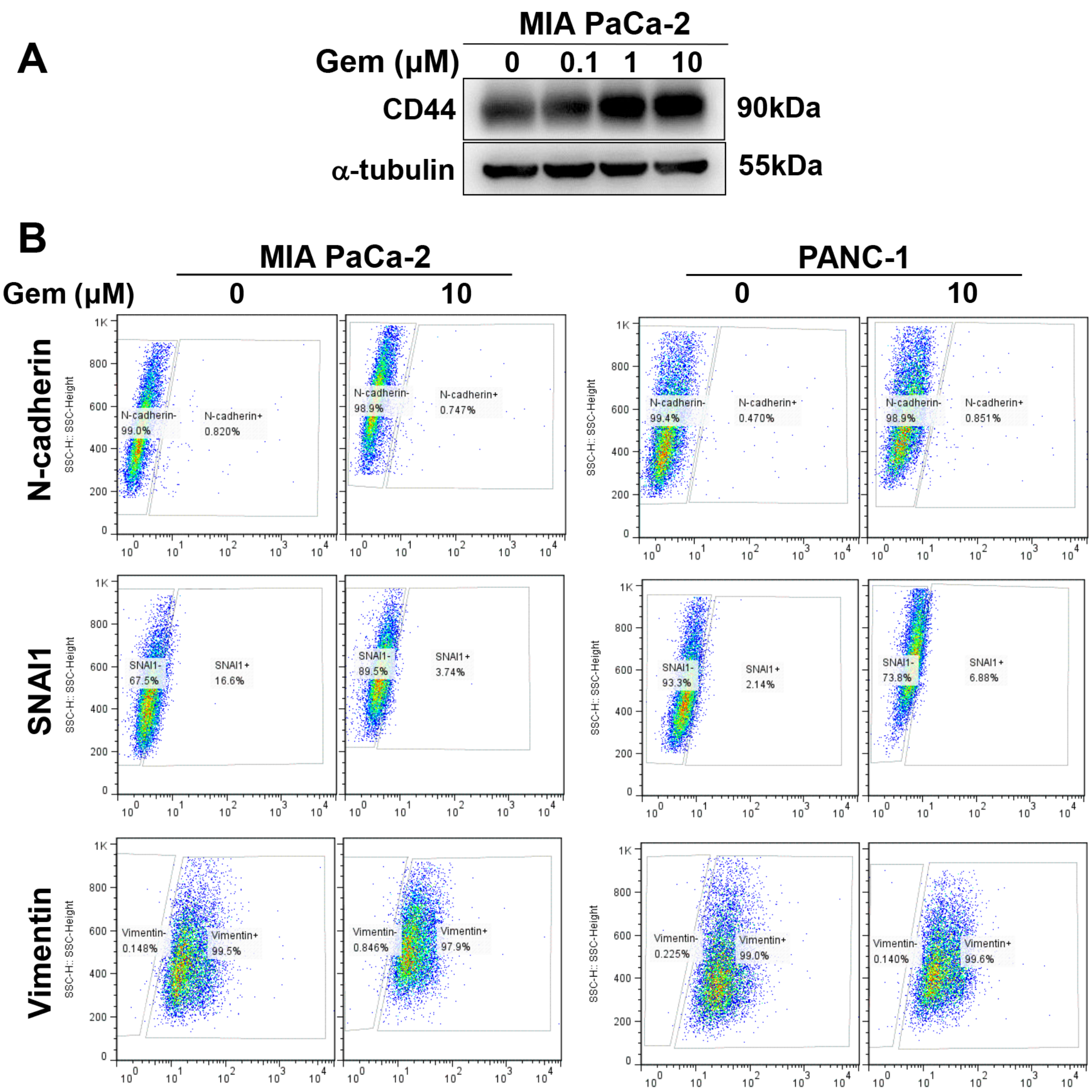


**Supplementary Figure S4: CD44 and EMT markers expression upon gemcitabine treatment. A.** Western blot of CD44 expression in pancreatic cancer cells treated with different doses of gemcitabine (0, 0.1, 1, or 10 μM) for 24 hours. **B.** Flow cytometry analysis of the EMT markers' (N-cadherin, SNAI1 and Vimentin) membrane levels in pancreatic cancer cells treated with gemcitabine (10 μM, 24 hours).
